# Whipple Procedure: A Five-Year Clinical Experience in Tertiary Care Center

**DOI:** 10.7759/cureus.11466

**Published:** 2020-11-13

**Authors:** Shabbar H Changazi, Qamar Ahmed, Samiullah Bhatti, Sumera Siddique, Eusha Abdul Raffay, Muhammad Waris Farooka, Mahmood Ayyaz

**Affiliations:** 1 Surgical Special Unit, Services Hospital Lahore, Lahore, PAK; 2 General Surgery, Services Institute of Medical Services, Lahore, PAK; 3 Surgery, Services Hospital Lahore, Lahore, PAK; 4 Surgical Unit 2, Services Hospital Lahore, Lahore, PAK; 5 General Surgery, Services Hospital Lahore, Lahore, PAK

**Keywords:** whipple procedure, pancreatic neoplasms, complications, mortality

## Abstract

Introduction: Whipple procedure is one of the major surgeries performed in tertiary care centers. Once considered a high mortality procedure is now being practicing with mortality declining to less than 5%. This study describes our five-year experience of the Whipple procedure in terms of preoperative, operative, and postoperative parameters of patients undergoing surgery in a local tertiary care setting.

Material and Methods: This was a non-randomized interventional study that was conducted at the Surgical Department of Services Hospital Lahore from January 2014 to December 2018. A total of 57 Whipple procedures were performed during this period. Demographic data, presenting symptoms, physical signs, past medical history, preoperative stenting details, intra-operative duration of surgery, postoperative course and complications, pathology, and causes of postoperative death were collected on a pre-designed questionnaire. Data were entered and analyzed by using SPSS 22 (IBM Corp., Armonk, USA).

Results: Out of 57 patients, 19 were females and 38 were males. The mean age of patients was 53±05 years. The most common presenting symptom was jaundice 39 (68.4%), followed by abdominal pain 32 (56.1%). The mean size of the tumor on CT-scan was 2.8±1.4 cm, the mean operation time was 315±38.3 min, mean blood loss during surgery was 500±130 ml, and mean hospital stay was 10±6 days. The major postoperative complication was the pancreatic fistula (12%). Twenty-one out of 39 patients presented with jaundice had undergone preoperative biliary stenting by endoscopic retrograde biliary stenting. The most common histological diagnosis was adenocarcinoma of pancreas 19 (33.3%). Out of 57 patients, nine (15.8%) patients expired in the first 30 days and the most frequent cause of mortality was septic shock.

Conclusion: In this study, the most common presentation of patients undergoing Whipple procedure was obstructive jaundice, the most frequent operative complication was pancreatic fistula, and the most prevalent histopathology was carcinoma of the pancreas. Perioperative parameters such as mean operative time, mean blood loss during surgery, and mean length of hospital stay were comparable with other studies. However, mortality in this study was slightly higher. It can be concluded that with meticulous surgical technique, securing hemostasis strictly and standard critical care postoperatively can decrease morbidity and mortality after the Whipple procedure.

## Introduction

Whipple procedure, also known as pancreaticoduodenectomy, is an imperative treatment for most neoplasms of the pancreas and malignant lesions of the peri-ampullary region of the duodenum. A standard Whipple procedure involves removal of the duodenum, proximal 15 cm of the jejunum, common bile duct, gall bladder, head of the pancreas, and a distal gastrectomy. Whipple et al. proposed the concept of pancreaticoduodenectomy for a peri-ampullary carcinoma in 1935 [1]. Subsequently, the pancreaticoduodenectomy became the operation of choice for patients with carcinoma of the pancreatic head, ampulla, distal bile duct, and duodenum. Initially, studies suggested a high operative mortality rate of the Whipple procedure, and surgery was abandoned [2,3]. Later on, several reports have suggested a sharp decrease in morbidity and mortality for the Whipple procedure. However, the morbidity rate remains quite high, approaching the 50% mark [4,5].

In recent practice, the Whipple procedure is a commonly performed procedure for benign and malignant neoplasms arising from the pancreas, peri-ampullary carcinomas, and cholangiocarcinoma. The most common complications are pancreatic fistula, delayed gastric emptying, hemorrhage, leakage of the hepatic and jejunum anastomosis, wound infection, and intraabdominal abscess, which affect mortality rate, hospitalization, and costs [6]. Adenocarcinoma of the pancreas accounts for 90% of all pancreatic carcinomas [7]. Considering cancer-related deaths, pancreatic carcinoma ranks at number 7 (worldwide), with an increased toll in developed countries [8]. Majority of the patients with localized pancreatic carcinoma have no recognizable symptoms that lead to late diagnosis, mostly after cancer has metastasized to other organs. Greater than 80% of patients have regional lymph nodes metastasis or distant metastases at the time of diagnosis [9]. The goal of this study was to evaluate the Whipple procedure from 2014 to 2018 in our hospital by analyzing the figures pertaining to the presenting signs and symptoms, postoperative clinical outcomes, and hospitalizations.

## Materials and methods

This was a non-randomized interventional study that was conducted at the Surgical Department of Services Hospital Lahore from January 2014 to December 2018. Ethical approval was obtained from the Institutional Review Board. Written informed consent was taken from all patients. A total of 60 patients were recruited in the study, however, three cases were excluded due to unresectability on exploration. Patients of both gender with age ranging between 20 and 70 years presenting with obstructive jaundice, weight loss, or vomiting with evidence of a mass in the pancreas on CT scan were included in the study. The patients with distant metastasis, superior mesenteric artery involvement, or extensive portal vein involvement were considered unresectable, hence excluded from the study. Patients with cardiorespiratory compromise or with uncontrolled diabetes were also excluded from the study (Video 1).

**Video 1:** Steps of the Whipple procedure

Preoperative evaluation included: detailed history and physical examination including abdominal examination along with ultrasound abdomen to evaluate the pancreas and biliary dilatation, biochemical tests to check serum bilirubin, alkaline phosphatase levels, CA 19-9 levels, and CT scan abdomen (with pancreatic protocol) to locate and confirm the location of the lesion. Before surgery, a single dose of antibiotics was administered intravenously and for deep venous thrombosis prophylaxis, low-molecular-weight heparin (Enoxaparin) was given subcutaneously. The abdomen was explored through rooftop incision and initial inspection for metastasis was done by finding ascites, liver metastasis, or advanced disease. Once metastasis was ruled out, kocherization of the duodenum was done. The dissection was carried out according to recommended protocols and respectability was assessed ensuring that the tumor was not encroaching the portal vein or superior mesenteric artery. Once the decision was made for formal resection, the head of the pancreas, duodenum common bile duct along with gallbladder and proximal 15 cm of jejunum was resected and anatomy was restored with triple anastomosis. All patients underwent duct to mucosa pancreatojejunostomy, mucosa to mucosa hepaticojejunostomy, and finally mucosa-to-mucosa gastrojejunostomy. The patients were advised for follow-up weekly for one month and then at monthly intervals up to one year and then six-monthly for three years.

Demographic data, presenting symptoms, physical signs, past medical history, preoperative stenting details, intra-operative duration of surgery, postoperative course and complications, pathology, causes of postoperative death, cause of re-exploration, and cause of readmission were collected on a pre-designed performa. Data were entered and analyzed by using SPSS 22. Quantitative data were presented by using mean ± SD. Qualitative data were presented by using frequency tables and percentages. This study was registered in clinicaltrial.gov (NCT03820583).

## Results

A total number of 60 procedures were performed from January 2014 till December 2018. However, three patients showed features of metastasis during exploration and the procedure was abandoned (only palliative procedure). As a result, a total of 57 patients was operated on for the Whipple procedure. Out of 57 patients, 19 were females and 38 were males. The mean age of patients was 53 ± 05 years. The most common presenting symptom was jaundice 39 (68.4%), followed by abdominal pain 32 (56.1%), and other symptoms in order of frequency like vomiting, fever, and weight loss (Table 1). The mean size of the tumor on CT-scan was 2.8±1.4 cm. In this study, the mean operation time was 315±38.3 minutes and the mean blood loss during surgery was 500±130 ml. The mean hospital stay length was 10±6 days. The major postoperative complication was the pancreatic fistula leak (12%) as depicted in Table 2. Preoperative biliary stenting through endoscopic retrograde cholangiopancreatography (ERCP) was done in 21 out of 39 patients, who presented with high jaundice >25 mg/dl. No other percutaneous biliary drainage was performed, unless it was clear that the tumor is advanced and irresectable. On histopathology 15 (26.3%), patients had benign pathology with serous adenoma of the pancreas being the common type while 42 (73.7%) patients had malignant disease with adenocarcinoma of the pancreas being the prevalent pathology (Table 3). Out of 57 patients, nine (15.8%) patients expired in the first 30 days and the most frequent cause of mortality was a septic shock (Table 4).

**Table 1:** Symptoms of patients

Presentation	No. of patients (n)	Percentage (%)
Jaundice	39	68.4
Abdominal pain	32	56.1
Vomiting	23	40.3
Fever	19	33.3
Weight loss	17	29.8

**Table 2:** Incidence of postoperative complications

Complications	No. of patients	Percentage (%)
Pancreatic fistula	07	12.3
Hemorrhage	06	10.5
Wound infection	04	7.0
Delayed gastric emptying	03	5.3
Enteric leak	01	1.7
Total	21	36.8

**Table 3:** Histological diagnosis of patients CBD: common bile duct.

Histological diagnosis		No. of patients	Percentage (%)
Benign		11	19.3
	Serious adenoma of the pancreas	7	12.3
	Non-specific fibrosis of pancreatic head	4	7
Malignant		46	80.7
	Adenocarcinoma of pancreas	17	29.8
	Solid pseudo-papillary carcinoma of the pancreas	13	22.8
	Cholangio-carcinoma of distal CBD	11	19.3
	Neuro-endocrine tumor	4	7
	Paraganglionoma	1	1.7

**Table 4:** Distribution of cause of mortality ARDS: acute respiratory distress syndrome.

Cause of mortality	No. of patients	Percentage(%)
Septic shock	5	8.78
Hemorrhage	3	5.3
Respiratory complication (ARDS)	1	1.7
Total	9	15.8

## Discussion

Whipple procedure is one of the major surgeries performed under the domain of general surgery. Once considered as a high mortality procedure is now being practiced in major hepatobiliary centers with mortality declining to less than 5%. However, the morbidity with the Whipple procedures is still significantly high. Pancreatic fistulae are considered as one of the most common short-term complications and ascending cholangitis is considered the most frequent long-term complication. Therefore, it is imperative to achieve enough skills to avoid these complications and diagnose them in an expeditious manner to treat the patient accordingly. This study describes our five-year experience of Whipple’s procedure in terms of preoperative, operative, and postoperative parameters of patients undergoing surgery.

In this study, 57 patients underwent the Whipple procedure for neoplasms of the pancreas, periampullary region, and duodenum. Among them 39 (68%) were males and 18 (32%) were females. The mean age of patients was 53 years and ranging from 35 to 70 years. The most common presenting symptom was jaundice and the mean size of the tumor on CT-scan was 2.8 cm. Among 39 jaundiced patients, 23 (8.9%) patients were having bilirubin levels greater than 21 mg/dl and had undergone preoperative biliary stenting by endoscopic retrograde biliary stenting. Although preoperative stenting decreased the bilirubin levels but increased the difficulty level as it increased operative time due to fibrosis of the periampullary region.

The mean duration of the Whipple procedure in the study was 315 minutes, which was slightly higher in a study done by Romano et al. [10] (295 mins); however, it was significantly lower than the study conducted by Saraee et al. [11] with a mean duration of 376 minutes. The mean blood loss during surgery was 500 ml and it was slightly higher than other studies [11]. The mean hospital stay length was shorter in this study as compared to other studies [10,11].

Whipple procedure is a very extensive operation with high morbidity. Common complications encountered after the surgery are pancreatic fistula, gastric emptying, hemorrhage, wound infection, and enteric leakage. In this study, pancreatic fistula formation was the most frequent complication accounting for 12.3% of patients. Halloran et al. [12] in a systemic review of 11 large centers described pancreatic fistula in 10.4% which is comparable with this study. However, the study conducted by Matsumoto et al. [13] showed pancreatic fistula in 4.2% of cases, and the study carried out by Romano et al. [10] observed pancreatic fistula in 4.12 of cases. The pancreatic fistula formation in these studies is lower as compared to the present study which can be attributed to surgical technique. In this study, 10.5% of patients developed hemorrhage which is higher as compared to other studies [12] which may be due to inadequate control at the time of surgery or due to slipped ligature. Wound infection occurred in 7% of cases which is comparable with other studies [12,13]. Finally delayed gastric emptying developed in 5.3% which is lower as compared to other studies [12,13]. Mortality in this study was higher as compared to other studies which can be attributed to a lack of advanced critical care services [10-14].

Histopathological data of the resected specimen showed that the most common histological finding was adenocarcinoma of pancreas followed by solid pseudo-papillary carcinoma and cholangio-carcinoma of CBD. Similar results were also found in other studies with pancreatic adenocarcinoma being the common indication of the Whipple procedure [10,11].

## Conclusions

The Whipple procedure is one of the major surgeries performed by hepatobiliary surgeons. Perioperative parameters such as mean operative time, mean blood loss during surgery, and mean length of hospital stay were comparable with other studies; however, mortality in this study was slightly higher. It can be concluded that with meticulous surgical technique, securing hemostasis strictly and standard critical care postoperatively can decrease morbidity and mortality after the Whipple procedure.

## References

[1] Whipple AO, Parsons WB, Mullins CR (1935). Treatment of carcinoma of the ampulla of Vater. Ann Surg.

[2] Strasberg SM, Drebin JA, Soper NJ (1997). Evolution and current status of the Whipple procedure: an update for gastroenterologists. Gastroenterology.

[3] Are C, Dhir M, Ravipati L (2011). History of pancreaticoduodenectomy: early misconceptions, initial milestones and the pioneers. HPB (Oxford).

[4] Lieberman MD, Kilburn H, Lindsey M, Brennan MF (1995). Relation of perioperative deaths to hospital volume among patients undergoing pancreatic resection for malignancy. Ann Surg.

[5] Suzuki Y, Fujino Y, Ajiki T, Ueda T, Sakai T, Tanioka Y, Kuroda Y (2005). No mortality among 100 consecutive pancreaticoduodenectomies in a middle-volume center. World J Surg.

[6] Karim SA, Abdulla KS, Abdulkarim QH, Rahim FH (2018). The outcomes and complications of pancreaticoduodenectomy (Whipple procedure): cross sectional study. Int J Surg.

[7] Feldmann G, Beaty R, Hruban RH, Maitra A (2007). Molecular genetics of pancreatic intraepithelial neoplasia. J Hepatobiliary Pancreat Surg.

[8] Rawla P, Sunkara T, Gaduputi V (2019). Epidemiology of pancreatic cancer: global trends, etiology and risk factors. World J Oncol.

[9] Wolfgang CL, Herman JM, Laheru DA, Klein AP, Erdek MA, Fishman EK, Hruban RH (2013). Recent progress in pancreatic cancer. CA Cancer J Clin.

[10] Romano G, Agrusa A, Galia M (2015). Whipple’s pancreaticoduodenectomy: surgical technique and perioperative clinical outcomes in a single center. Int J Surg.

[11] Saraee A, Vahedian-Ardakani J, Saraee E, Pakzad R, Wadji MB (2015). Whipple procedure: a review of a 7-year clinical experience in a referral center for hepatobiliary and pancreas diseases. World J Surg Oncol.

[12] Halloran CM, Ghaneh P, Bosonnet L, Hartley MN, Sutton R, Neoptolemos JP (2002). Complications of pancreatic cancer resection. Dig Surg.

[13] Matsumoto Y, Fujii H, Miura K (1992). Successful pancreatojejunual anastomosis for pancreatoduodenectomy. Surg Gynecol Obstet.

[14] Yeo CJ, Cameron JL, Lillemoe KD (1995). Pancreatoduodenectomy for cancer of the head of the pancreas: 201 patients. Ann Surg.

